# Using art and history to communicate immunology to a broad audience

**DOI:** 10.1038/s41577-024-01090-w

**Published:** 2024-09-16

**Authors:** Francesca Di Rosa

**Affiliations:** Institute of Molecular Biology and Pathology, National Research Council of Italy (CNR), Rome, Italy; Francis Crick Institute, London, UK

## Introduction

Can vaccination be portrayed as a ballet? This and other unconventional ideas formed part of an outreach project called “Vaccination, a time machine”, which was exhibited in July 2024 at the Royal Society Summer Exhibition, a science festival that showcases cutting-edge research in the UK. The display was designed to communicate basic concepts about vaccine-elicited immunity in the context of recent and ongoing results regarding T cell responses to vaccine boosters, and immuno-monitoring of patients with COVID-19 in pre- and post-vaccination settings.

For this project, we paid particular attention to the use of multidisciplinary modalities, specifically art and history, in the setting of a bespoke architect-designed stand. Our aim was to communicate scientific information and elicit surprise, whilst introducing visitors to the past and future of vaccination. Importantly, given the audience varied hugely in age, interests and subject knowledge, we offered various different ways to navigate the exhibit during the visit. A leaflet entitled “Senses of time” and online contents were available as take-home materials.

With a focus on “time” as a keyword, the exhibit explored concepts and challenges related to vaccination, such as how to achieve long-term immunity and when to administer additional vaccine boosters. The title of our exhibit provoked visitors to think of vaccination as an imaginary apparatus that propels people into a healthy future by accelerating immune responses. Our stand offered non-digital educational activities with underlying scientific messages, that were created in-house at the Francis Crick Institute (by Adrian Hayday, Ambra Natalini, and I, immunologists, together with Kat Nilsson, a public engagement professional, and Albane Imbert and Christina Dix, who designed and realized the objects). Thus, customized hourglasses simulated the impact of time on immunity and a modified version of the children’s game “Kerplunk” mimicked the expansion of antigen-specific lymphocytes after a booster dose of vaccine. Young scientists guided the “hands-on” educational activities and senior immunologists were ready to discuss state-of-the-art research and ideas behind the exhibit.

A welcoming stand (designed by the architect Fabrizio Lepore) facilitated an immersive visitor experience and honored the unique history and tradition of the Royal Society, for example by emphasizing a bookcase in the backdrop containing the permanent collection of the Philosophical Transactions, the world’s first recorded scientific journal (launched by the Royal Society in 1665). The stand had display cases for both artworks and historical documents, and a two-level plinth resembling a small-scale sculpture that held the objects for interactive activities and allowed access for children, adults, and persons using wheelchairs. We presented original documents about “variolation” from the 18^th^ century (selected from the Royal Society library and archives in collaboration with the historian Louisiane Ferlier) and displayed three arresting glass sculptures of Variola Virus, Human Immunodeficiency Virus (HIV), and an imaginary future virus mutant, by the British artist Luke Jerram.

One of the biggest attractions was a beautiful short film (by Tim Whitehead and Michael O’Halloran) representing a dance that was created by the Royal Academy of Dance for our exhibit. Under the guidance of the choreographer Dennie Wilson, dancers acting as “viruses”, “lung cells”, “lymphocytes”, and a “vaccine” evoked the dynamics of infection and immunity. At the opening of the exhibit, Francesco Seganfreddo, a philosopher of science and science communicator, launched a podcast miniseries, entitled “On time”. The first episode featured an imaginary conversation between Lady Mary Wortley Montagu and Emmanuel Timoni, two historical figures in immunology of the early 1700s. Future episodes will explore topics that include memory and speed, and their relationships to immunology, and feature scientists, artists, and historians.

## The “5C” formula

Looking back, I extrapolate five leading principles, here called the “5Cs”, from this project and the responses we received from our colleagues and the public. The “5Cs” are likely to be valuable also for other types of science outreach projects, as they can act as a framework to facilitate scientists and non-scientists alike to connect with the relevance of scientific concepts to our everyday lives.

### Concept

the scientific content guided the choice of contributions from art and history. In our project, the core of the exhibit was the notion of immunological memory induced by vaccination. This included individual variation in response to vaccination, and the potential loss of memory over time. In a broader sense, envisioning the scientific content as a wheel hub and the different display modalities as spokes is a strategic concept to reinforce the outreach message. This approach can strengthen the learning process for visitors who have widespread interests and facilitate a full immersion, while still conveying the key points to those only attracted by one type of presentation.

### Collaboration

both scientists and contributors from other fields developed creative ideas for the project, and planning was refined in a highly interactive manner. In our project, we were able to fuse Immunology, Design, Architecture, History, Communication, Contemporary Art, and Dance, owing to the interactions within our team and with external collaborators. Multidisciplinary collaboration of this type nurtures the thought-provoking integration of creative inputs. A further advantage is that ideas with regards to science communication can be immediately tested on non-scientific members of the team.

### Coherence

this was achieved at multiple levels. Regarding the scientific content, heterogeneous inputs were harmonized to communicate a consistent message. The various means employed to engage the public were all optimized to complement each other. The display was coherent with the historical collections and the site where the exhibit was displayed. In our project, the use of “time” as keyword was a crucial instrument to maintain cohesion across all the different parts of the exhibit. Overall, coherence can be a big challenge for a multidisciplinary communication project. The goal is achieved when visitors feel they can connect the dots of the ‘exhibit puzzle’, rather than being disoriented by arbitrary stimuli.

### Conversation

visitors were encouraged to talk to team members by a welcoming and attractive environment that provoked questions and elicited curiosity. We invited visitors to freely navigate the exhibit according to their inclinations, while still offering them some light-touch orientation. In my opinion, a spontaneous approach to exhibits’ contents can more easily nurture critical thinking than structured educational activities, such as formal talks.

### Continuity

the exhibit experience was not finished on the closing day, but it continued with a legacy. In our project, the exhibit generated a lively exchange of personal comments, emails, messages on social platforms, that is still ongoing, thanks also to the attention given to the exhibit in the press and the general media. Additional episodes of our podcast miniseries “On time” are currently in production. While offering the opportunity to further delving into the exhibit subject, and even to listen to the podcast as a stand-alone material, we hope also to receive advice and criticism that will be useful for future outreach plans.

## Concluding thoughts

A multifaceted display including art and history is likely to make the scientific concepts linked to the exhibit experience easier to remember, in agreement with the emerging concept of moving from STEM (science, technology, engineering, and mathematics) to STEAM (science, technology, engineering, art and mathematics) to engage the public. Personally, I also found that leaving my immunology comfort zone to find connections with other disciplines resulted in a new mindset, with a cascade of positive consequences. In the exhibit development phase, collaborating with experts from other fields influenced the design of the outreach project. For example, both the sculptures and the choreography were essential to propose a fascinating view of the interaction between viruses and the immune system, by contrast to the more commonplace analogy with a war. In the delivery phase, the stand offered an aesthetic experience that people could enjoy regardless of their scientific proficiency. This appeared to put visitors and members of the scientific team at ease. It also facilitated two-way communication, drastically decreasing the risk of an overly didactic approach.

Obviously, an outreach project of this type extends beyond the boundaries of a classical educational activity. Considering that non-conventional displays that include humanities can help to embed science, art and history in society, it is desirable that economic support for this kind of activities is increased, e.g., by interdisciplinary funding bodies from government, academic institutions, no-profit organizations, charities, etc. Such investments are of high priority to build scientific literacy in the public. More specifically, vaccine-related issues are current hot topics in the public debate after COVID-19 pandemic and rapid development of anti-SARS-CoV-2 vaccines. Fostering multidisciplinary outreach projects centered on vaccination and closely related subjects will be important to overcome and/or prevent obstacles in the public understanding of immunology.

## Hyperlinks (in order of appearance in the text)

“**Vaccination, a time machine**”
https://www.crick.ac.uk/research/labs/adrian-hayday/vaccination-a-time-machine

**“On time”**

https://www.crick.ac.uk/research/labs/adrian-hayday/vaccination-a-time-machine/vaccination-a-time-machine-podcast

